# Painless destructive thyroiditis in a patient with resistance to thyroid hormone: a case report

**DOI:** 10.1186/s13044-019-0072-2

**Published:** 2019-10-25

**Authors:** Tomoko Nagamine, Jaeduk Yoshimura Noh, Naoya Emoto, Takahito Kogai, Akira Hishinuma, Fumitaka Okajima, Hitoshi Sugihara

**Affiliations:** 1 Department of Endocrinology, Diabetes and Metabolism, Graduate School of Medicine, Nippon Medical School, Tokyo, Japan; 2 Department of Internal Medicine, Ito Hospital, Tokyo, Japan; 3 Department of Infection Control and Clinical Laboratory Medicine, Dokkyo Medical University, Tochigi, Japan

**Keywords:** Thyroid, Resistance to thyroid hormone, Painless thyroiditis, Scintigraphy

## Abstract

**Background:**

Resistance to thyroid hormone (RTH) usually features a syndrome of inappropriate secretion of thyroid-stimulating hormone (SITSH) without suppression of the typical high thyroid hormone levels. However, some patients with RTH show thyroid-stimulating hormone (TSH) suppression due to thyrotoxicosis. We report a case of painless thyroiditis in a patient with RTH that was misdiagnosed as Graves’ disease because of TSH-suppressed thyrotoxicosis.

**Case presentation:**

A sixteen-year-old boy consulted a local general physician for fatigue. He had a goiter, and biochemical analysis showed TSH < 0.1 μIU/mL, free triiodothyronine (FT3) of 2.70 pg/mL, and free thyroxine (FT4) of 3.6 ng/dL. He was diagnosed with Graves’ disease and was treated with 20 mg thiamazole. One year later, he was referred to the department of endocrinology because of SITSH. He was finally diagnosed with RTH due to the finding of a heterozygous missense mutation (methionine 334 threonine) in the thyroid hormone receptor β gene. Three years after cessation of thiamazole, his hyperthyroxinemia showed marked exacerbation with TSH suppression. We diagnosed him with painless destructive thyroiditis because of low technetium-99 m (Tc-99 m) uptake in the thyroid. Extreme hyperthyroxinemia was ameliorated, with a return to the usual SITSH levels, within 1 month without any treatment.

**Conclusion:**

The present case demonstrates that diagnosing RTH is difficult when patients show hyperthyroxinemia with complete suppression of TSH to undetectable levels, and the data lead to misdiagnosis of RTH as Graves’ disease. The initial diagnosis is important, and Tc-99 m scintigraphy is useful for the differential diagnosis of thyrotoxicosis accompanying RTH.

## Background

RTH is an inherited syndrome of reduced tissue responsiveness to thyroid hormone [1–3]. RTH is characterized by elevated serum levels of FT4 or FT3 in the presence of high normal or slightly increased serum TSH concentrations. However, several reports have demonstrated that TSH is suppressed to very low or undetectable levels in patients with RTH coexisting with Graves’ disease [4–8]. In these cases, a correct diagnosis is difficult if patients are not diagnosed with RTH beforehand. TSH levels may also be suppressed in patients with RTH in cases of thyrotoxic diseases other than Graves’ disease. In this report, we present a patient with RTH who suffered from painless thyroiditis and whose TSH was completely suppressed to undetectable levels during transient hyperthyroxinemia.

## Case report

**Fig. 1:**
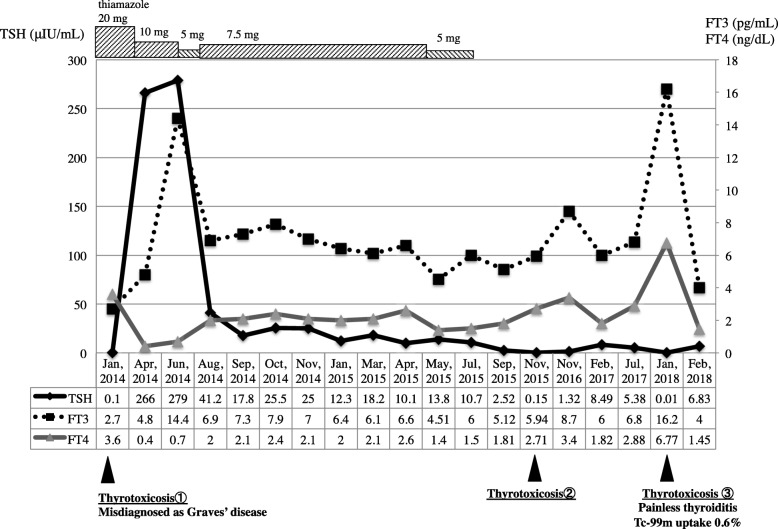
Time course of thyroid function. The first thyrotoxicosis episode was misdiagnosed as Graves’ disease and rapidly changed to a hypothyroid state due to thiamazole administration. One and a half years later, a second thyrotoxicosis episode occurred, and the patient recovered without treatment. Four years after the first episode of thyrotoxicosis, the third thyrotoxicosis episode occurred. Low Tc-99 m uptake without fever and pain indicated painless thyroiditis. Retrospectively, all of these thyrotoxicosis episodes seemed to be repeated painless thyroiditis.

A 16-year-old male with a goiter was referred to Nippon Medical School in May 2015. His medical history was unremarkable until 14 years of age (2013). His father and grandmother had hearing impairments. One year before the referral, the patient had complained of fatigue and a goiter and had consulted a family practitioner. A thyroid function test showed TSH < 0.1 μIU/mL, FT3 of 2.70 pg/mL, and FT4 of 3.6 ng/dL. The doctor misdiagnosed this thyrotoxicosis episode as Graves’ disease without performing an ultrasound examination or confirming the presence of antithyroid antibodies and treated the patient with thiamazole 20 mg. Three months later, the patient showed hypothyroidism (TSH 265.7 μIU/mL and FT4 0.4 ng/dL) (Fig. 1), and the doctor reduced the thiamazole dose. However, SITSH (TSH 12.3–18.2 μIU/mL and FT4 2.0–2.1 ng/dL) continued for 1 year, and the patient was referred to our hospital with a treatment plan of thiamazole at 5 mg and 10 mg doses alternating every other day. His thyroid function was indicated by TSH of 13.64 μIU/mL, FT3 of 4.51 pg/mL, and FT4 of 1.41 ng/dL. We considered his SITSH to be a phenomenon of the transition from hypothyroidism to hyperthyroidism, and we thus decreased the thiamazole to 5 mg. His SITSH continued for the next 3 months, and magnetic resonance imaging (MRI) showed pituitary swelling. Because we could not exclude the possibility that the patient had TSHoma, we stopped thiamazole and admitted him to our ward for differential diagnosis of SITSH.

**Fig. 2:**
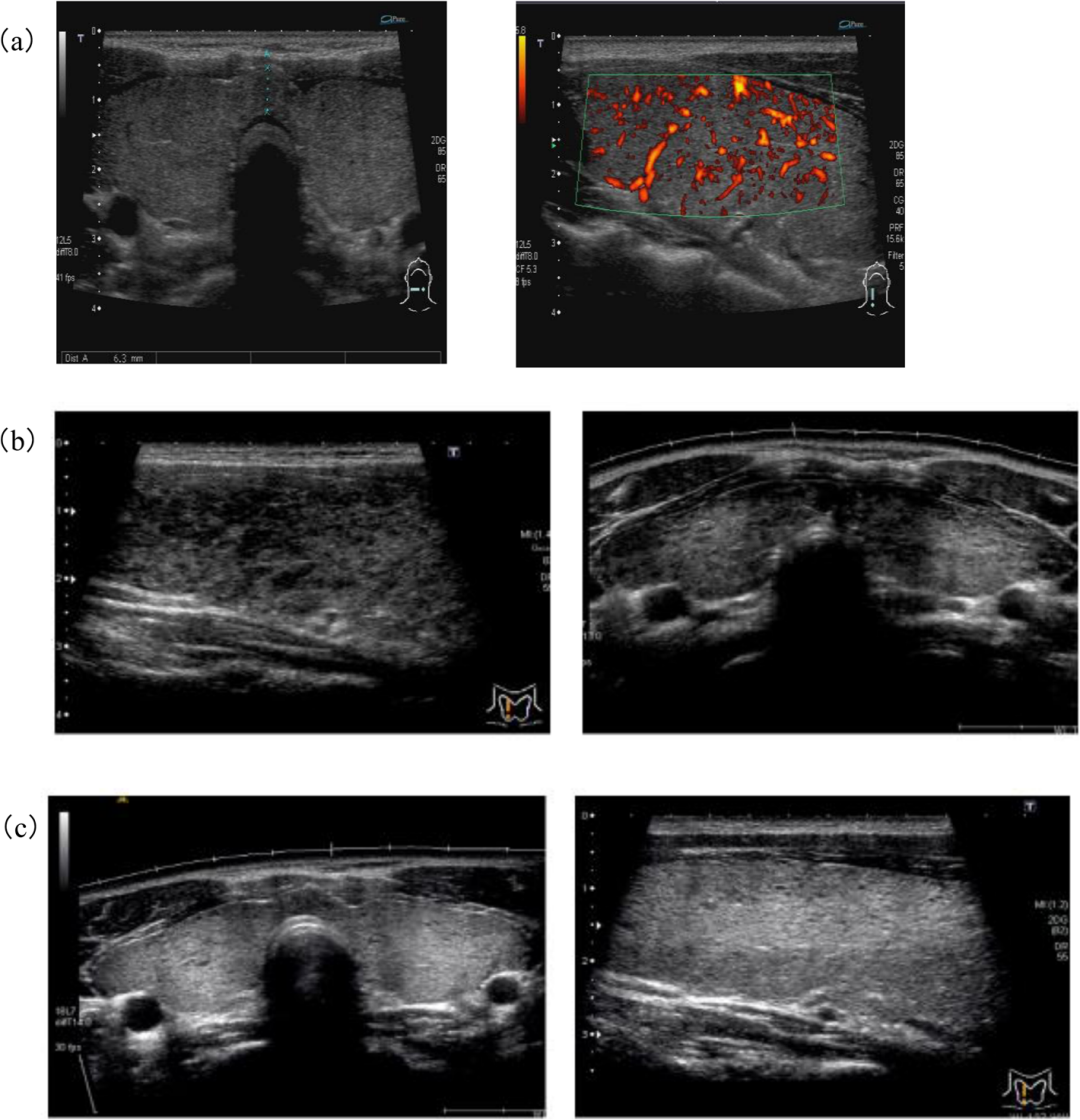
Thyroid ultrasonography in each phase: (**a**) the SITSH state, while the patient was hospitalized in August 2015; (**b**) the third thyrotoxicosis episode in January 2018; and (**c**) recovery from painless thyroiditis in May 2019. (**a**) Moderate goiter, slightly heterogeneous hypoechogenicity, and moderately rich thyroid blood flow were detected. (**b**) There were focal hypoechoic areas without tenderness. (**c**) The hypoechoic area became invisible, and the echogenicity and homogeneity returned to the original condition

The patient’s height was 158 cm, and his body weight was 52.3 kg. His temperature was 36.9 degrees, his heart rate was 83 beats per minute, and his blood pressure was 128/73 mmHg. In his usual SITSH state, thyroid ultrasonography showed a moderate goiter, slightly heterogeneous hypoechogenicity, and moderately rich blood flow (Fig. 2a). TSH receptor antibodies (TRAb), thyroid-stimulating autoantibodies (TSAb), and thyroid-peroxidase antibodies (TPO-Ab) were all negative, and only anti-thyroglobulin antibodies (Tg-Ab) were positive. The thyrotropin-releasing hormone test showed a normal TSH response, and the octreotide and bromocriptine test did not suppress the TSH levels. Sex hormone-binding globulin (SHBG) was within the normal range. These data suggested that his SITSH was unlikely to be due to TSHoma. Then, we examined whether the patient’s family members had SITSH, and we identified SITSH in his father and his older brother. We found a genetic mutation of thyroid hormone receptor-beta (TRβ) exon 9, methionine 334 threonine, which was the same mutation reported by Mannavola et al. [9, 10], and we diagnosed the patient with RTH.

He developed a second case of thyrotoxicosis in November 2015, but it was mild and short. For the next two and a half years, his condition was stable as SITSH.

In January 2018, he complained of tachycardia and fatigue caused by a third episode of thyrotoxicosis. His TSH was suppressed, and TRAb remained negative. The technetium-99 m (Tc-99 m) thyroid uptake was 0.6%, which was low compared to his usual state (9.7%). Therefore, we diagnosed this third episode of thyrotoxicosis as painless destructive thyroiditis. Thyroid ultrasonography during the third episode of thyrotoxicosis detected a moderate goiter, slightly heterogeneous hypoechogenicity, and moderately rich thyroid blood flow (Fig. 2b). The patient’s thyroid function returned to his usual SITSH state without any treatment within 1 month. The hypoechoic area became invisible, and echogenicity and homogeneity returned to the original condition in January 2019, 16 months after the last episode of painless thyroiditis (Fig. 2c).

## Discussion

We presented a case of repeated thyrotoxicosis with RTH. RTH usually features SITSH without suppression of the typical high thyroid hormone levels. However, some patients with RTH show TSH suppression during thyrotoxicosis episodes. Several reports have described patients with RTH complicated by thyrotoxic diseases, such as Graves’ disease [4–8]. TSH levels can also be suppressed in patients with RTH in combination with thyrotoxic diseases other than Graves’ disease. Taniyama et al. reported toxic multinodular goiter in patients with RTH [11] and episodes of repeated painless thyroiditis [12].

Painless thyroiditis usually occurs as a form of autoimmune thyroid disease, and the prevalence rate of thyroid autoantibodies is 2.36 times higher in RTH patients than in controls [13]. Furthermore, painless thyroiditis is more common in Japan than in the United States or Europe. We previously reported that the incidence of transient hyperthyroxinemia with suppressed Tc-99 m in hyperthyroid disease was 14.7% in a local area in Japan [14]. Other reports have shown that the incidence of painless thyroiditis was 10% in patients with hyperthyroid disease in Japan but was less than 1% in Denmark [15, 16]. These results suggest that we must consider painless thyroiditis in RTH patients with thyrotoxicosis, especially in Japan. In our present case, a previous doctor misdiagnosed the first episode of thyrotoxicosis as Graves’ disease, but painless thyroiditis could be considered as a differential diagnosis in terms of its frequency and the low FT3/FT4 ratio.

We think of these thyrotoxicosis episodes as repeated painless thyroiditis for four reasons. First, TRAb was negative at all times. Second, treatment with antithyroid drugs was nontherapeutic and caused severe hypothyroidism during the first episode of thyrotoxicosis. Third, the FT3/FT4 ratio was low during all episodes of thyrotoxicosis. Finally, the Tc-99 m thyroid uptake was low, and the absence of fever or tenderness was not typical for subacute thyroiditis.

It is difficult to diagnose thyrotoxicosis with RTH. In general practice, thyroid scintigraphy is not available; therefore, the differential diagnosis of Graves’ disease and other disorders depends on the existence of TRAb. Thyroid ultrasonography also provides useful data on thyroid size, tenderness, homogeneity, and blood flow. It was reported that thyroid blood flow is significantly higher in Graves’ disease than in destructive thyroiditis. A thyroid blood flow of less than 4% suggests destructive thyroiditis [17]. In the present case, thyroid ultrasound was not performed during the first episode of thyrotoxicosis. Thyroid ultrasonography revealed a focal hypoechoic area during the third episode of thyrotoxicosis, and the hypoechoic region became invisible after recovery (Fig. 2b, c). There were no data on thyroid blood flow during thyrotoxicosis, but these changes in echogenicity suggested thyrotoxicosis after the destruction and regeneration of the thyroid.

When the patient was hospitalized in his usual SITSH state, his thyroid blood flow was relatively rich and was similar to that in mild Graves’ hyperthyroidism. Because there are no sufficient thyroid ultrasonography data for RTH, especially in the thyrotoxic state, thyroid ultrasounds seem to lack sufficient information for discriminating Graves’ disease from other disorders. Moreover, TRAb can be positive in destructive thyroiditis. Therefore, only radioiodine or technetium uptake can provide a correct diagnosis of thyrotoxicosis with RTH. Although it is difficult to determine the presence of RTH when patients show hyperthyroxinemia with complete suppression of TSH to undetectable levels, the initial diagnosis is important.

Compared with single episodes of thyroiditis, repeated painless thyroiditis is characterized by male gender, younger age, positive thyroid autoantibodies, and higher peak FT3 and FT4 levels [18]. Thyroiditis can occasionally develop in clusters in nursery school workers, and thus, an unidentified virus was hypothesized to be a cause of thyroiditis [19]. Because our patient always developed thyrotoxicosis in winter, some viral infection or seasonal allergy might have been a trigger. The association between RTH and repeated painless thyroiditis was unclear, but chronic thyroiditis, which often coexists with RTH, affected the patient’s tendency toward painless thyroiditis.

## Conclusion

We reported a case of repeated painless thyroiditis in a patient with RTH. RTH is known as a cause of SITSH and is rarely discovered in patients with thyrotoxicosis. It is difficult to treat thyrotoxicosis with RTH because these patients have FT3 and FT4 levels that are higher than normal. Therefore, the initial diagnosis is important during TSH suppression. Tc-99 m scintigraphy can detect the clinical condition of thyrotoxicosis regardless of whether the patient has concomitant RTH.

## Data Availability

Data sharing is not applicable for this article because no datasets were generated or analyzed during the current study.

## Abbreviations

FT3: Free triiodothyronine
FT4: Free thyroxine
MRI: Magnetic resonance imaging
RTH: Resistance to thyroid hormone
SHBG: Sex hormone-binding globulin
SITSH: Syndrome of inappropriate secretion of thyroid-stimulating hormone
Tc-99 m: Technetium-99 m
Tg-Ab: Anti-thyroglobulin antibodies
TPO-Ab: Thyroid-peroxidase antibodies
TRAb: TSH receptor antibodies
TRβ: Thyroid hormone receptor-beta
TSAb: Thyroid-stimulating autoantibodies
TSH: Thyroid-stimulating hormone
